# Managing Menopausal Skin Changes: A Narrative Review of Skin Quality Changes, Their Aesthetic Impact, and the Actual Role of Hormone Replacement Therapy in Improvement

**DOI:** 10.1111/jocd.70393

**Published:** 2025-08-23

**Authors:** Bianca Viscomi, Mariana Muniz, Sonja Sattler

**Affiliations:** ^1^ Dermatology São Paulo Brazil; ^2^ Private Clinic for Aesthetic Dermatology, Aesthetic Dermatologic Surgery and Plastic Surgery Darmstadt Germany

**Keywords:** estrogen, hormone replacement therapy, menopause, perimenopause, skin health, skin quality

## Abstract

**Background:**

The decline in estrogen during menopause contributes to structural and functional skin changes, including decreased collagen production, reduced elasticity, and moisture loss, resulting in dryness and wrinkling. Today, hormone replacement therapy (HRT) enhances skin quality by promoting collagen synthesis, elasticity, and hydration. Minimal invasive aesthetic treatment could add value to this patient group.

**Methods:**

To assess the relationship between menopause, hormones, and HRT on skin health, we conducted a comprehensive literature search using the terms (menopause OR menopausal) AND (aging OR aging) AND (skin) AND (estrogen OR estrogen OR hormones) AND (hormone replacement therapy OR HRT). To explore aesthetic treatments specific to menopausal women, searches included (“menopause” OR “menopausal”) AND (“aesthetics” OR “aesthetics” OR “cosmetic” OR “filler”). Additional studies were identified from references in the uncovered articles.

**Results:**

We reviewed the impact of hypoestrogenism and HRT on menopausal skin and identified unmet needs in its aesthetic management. Most studies reported positive associations between HRT and improvements in menopausal skin aging, but findings were sometimes inconsistent. Despite decades of research, clinical guidelines do not support HRT use solely for estrogen‐deficient skin due to a lack of robust clinical trials on skin‐specific therapy. Many women remain unaware of the skin‐related impacts of menopause, which can affect quality of life, and few aesthetics studies analyze data based on menopausal status or HRT use. Given the growing demand for aesthetic treatments in this population, future studies should intentionally incorporate demographic data and analyses on menopausal status and HRT use.

## 1 Introduction

Aging leads to predictable changes in the skin [1, 2, 3], connective tissue, subcutaneous fat tissue, and bone of the face and body, influenced by factors like genetics, ethnicity, smoking, and sun exposure [4, 5]. In soft tissue, these changes arise from the gradual loss and disorganization of collagen and elastic fibers, contributing to the appearance of an aged face and body with increasing alterations in density, moisture levels, texture, and skin tone [6, 7]. These changes can negatively impact self‐esteem, as skin appearance strongly impacts emotional well‐being, self‐image, life satisfaction, and social interactions [8, 9, 10, 11].

In addition to these gradual changes, shifts in hormones, specifically those which accompany menopause, can cause significant changes in skin appearance within a comparatively short period of time. The perimenopausal years are marked by an accelerated decline in skin quality, largely due to declining estrogen levels, which impair collagen production and the integrity of the extracellular matrix (ECM) [8, 12, 13]. Estrogen replacement can diminish many of these effects by enhancing the content and quality of collagen, increasing vascularization, and improving epidermal hydration, elasticity, and thickness [14]. However, the precise biological mechanisms by which hormonal fluctuations during menopause influence these aging processes remain poorly understood [13]. Furthermore, clinical guidance is limited for specifically managing peri‐ and postmenopausal women seeking aesthetic treatments to improve skin quality [8].

Given that women spend roughly one‐third of their lives in the postmenopausal phase, it is clinically valuable to understand and mitigate the impact of these hormonal changes to improve patient outcomes [8]. Furthermore, the influence of hormones on treatment efficacy is poorly understood. To address these gaps in understanding, we conducted a comprehensive literature search on how menopause, hormones, and hormone replacement therapy (HRT) affect skin quality. This review explores the effects of hypoestrogenism on the skin, evaluates the impact of HRT, and identifies unmet needs that, if addressed, could guide the aesthetic management of menopausal women. In this review, we use “skin quality” to refer to measurable features such as elasticity, hydration, thickness, and texture; “skin health” to encompass both these structural properties and physiological function; and “skin aging” to describe age‐related structural and functional changes, particularly those accelerated by estrogen loss.

## 2 Methods

To comprehensively explore the relationship between menopause, skin quality, and HRT, alone or along with aesthetic treatment options, a systematic literature search was conducted across multiple databases, including PubMed, Google Scholar, Scopus, and the Cochrane Library. Publications from 1971 through 2024 were included to capture both pioneering research and current consensus on treatment approaches.

The search strategy employed the following terms: (menopause OR menopausal) AND (aging OR aging) AND (skin) AND (estrogen OR estrogen OR hormones) AND (hormone replacement therapy OR HRT). To further explore aesthetic treatments for menopausal women, searches were conducted using (“menopause” OR “menopausal”) AND (“aesthetics” OR “aesthetics” OR “cosmetic” OR “filler”). Additional relevant studies were identified through a manual review of the references cited in the included articles.

## 3 Results

Aging skin is marked by structural changes, leading to thinning, dryness, scaliness, wrinkling, and loss of elasticity, along with functional changes such as reduced barrier function, slower wound healing, and impaired immune and thermoregulatory responses [15, 16]. In women, many transformations characteristic of aging skin emerge alongside the decline in estrogen during the transition to menopause [16, 17, 18, 19, 20]. While aging‐related skin changes develop gradually in men, those associated with menopause can be abrupt and distressing for women [13, 21, 22].

Menopause marks the end of a woman's reproductive life, defined by the permanent cessation of menstruation for 12 consecutive months due to the loss of ovarian function, which can occur naturally with age or be induced by surgery, chemotherapy, or radiation [8, 23]. Although menopause has a clear definition and is marked by the complete cessation of ovarian hormonal production, it is important to note that changes and fluctuations in hormonal production start 2–8 years earlier—a transitional period known as perimenopause [8, 23, 24]. The final menstrual period usually occurs before 58 years of age, with occurrence before the age of 40 considered premature [23]. The exact timing of menopause is influenced by genetic factors and environmental elements, including UV exposure and smoking, as well as lifestyle factors such as diet, alcohol consumption, stress, and sleep patterns [8, 23]. Menopausal symptoms arise from hormonal changes, primarily driven by a sharp decline in ovarian secretion of estrogens, androgens, and progesterone, which are manifested in the skin as reduced epidermal and dermal thickness, lower collagen content, decreased elasticity, and increased dryness and fragility [16, 17, 18, 19, 20, 22, 25].

### 3.1 Impact of Hypoestrogenism on Skin

**FIGURE 1:**
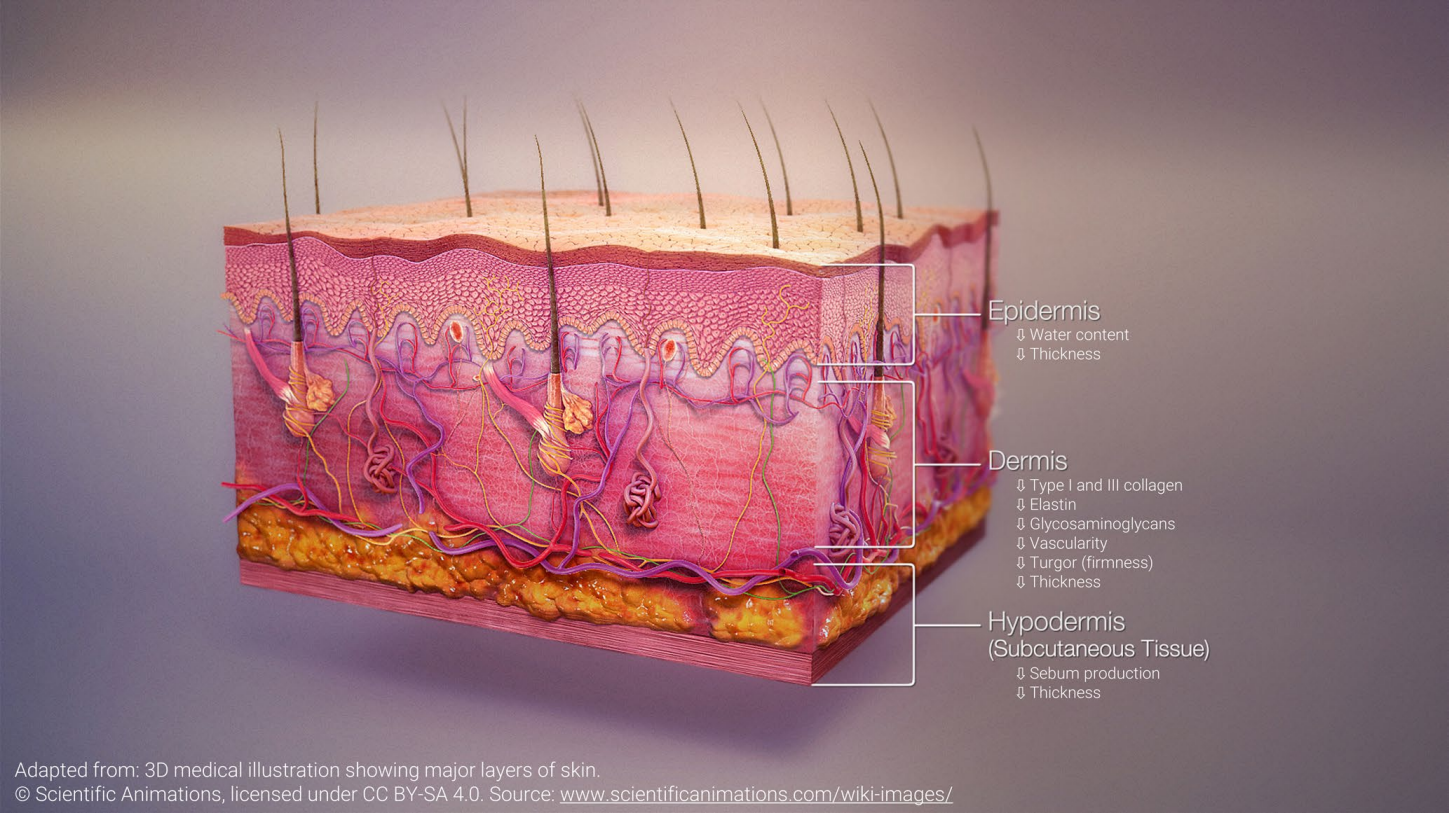
Effects of estrogen deficiency on skin structure. Effects of estrogen deficiency on skin structure: Reduced type I and type III collagen in the ECM decrease structural integrity and elasticity, contributing to skin thinning and increased rigidity. Loss of elastin, hydrophilic glycosaminoglycans, and sebum production results in dryness, reduced moisture retention, and increased skin rigidity, leading to wrinkling. Reduced vascularity impairs wound healing and weakens the skin barrier.

The skin functions as an endocrine organ, with its cells both producing and responding to estrogen and other hormones, making it a key target for hormonal regulation [8, 12]. Adequate levels of hormones, primarily estrogen, 17β‐estradiol, are required for the skin's optimal structural integrity and functional capacity [26, 27]. After menopause, estrogen production from the ovaries ceases, shifting the burden to peripheral tissues like the skin, where aromatase activity converts dehydroepiandrosterone (DHEA) into estrogens [26, 28]. Specifically, peripheral tissues convert DHEA into estradiol and estrone post‐menopause, with trace production of estriol, a weaker estrogen that is more prominent during pregnancy. All three act through estrogen receptors, with estrogen receptor β (ER‐β) being the predominant receptor subtype in the skin [14, 29]. With age‐related decline in DHEA, the skin's ability to maintain estrogen levels decreases, contributing to post‐menopausal estrogen deficits [26]. The scarce availability of estrogen in aging skin is reflected in the reduced levels of estrogen receptors in the skin following menopause [16, 30]. Thus, as surmised by Merzel Šabović et al. [28], three underlying mechanisms drive menopausal skin changes: reduced systemic estrogen levels due to diminished ovarian synthesis, lower local estrogen production within the skin, and decreased expression of estrogen receptors in the skin. The resulting hypoestrogenism leads to skin thinning, atrophy, reduced collagen, decreased elasticity, and reduced vascularity (Figure 1) [14, 28]. Dryness results from the loss of hydrophilic glycosaminoglycans, contributing to a direct reduction in water content and skin turgor [27]. Together, these changes contribute to wrinkling, a weakened skin barrier, and impaired wound healing [12, 14, 28].

#### 3.1.1 Collagen Content, ECM Stability, and Skin Thickness

Observational and histologic studies by Brincat et al. [20, 31] demonstrated that skin collagen content declines with menopausal age rather than chronological age, at an average rate of 2.1% per postmenopausal year over a 15‐year period. This reduction includes both type I collagen, which provides tissue strength, and type III collagen, which contributes to the skin's elastic properties [32]. In parallel, menopausal aging is associated with a weakening ECM, further contributing to the gradual thinning of the skin over time, measured at approximately 1.13% per year during the first 19 years postmenopause [31]. Changes in skin viscoelasticity also occur, with declines in elasticity at 1.5% per year, accompanied by increased distensibility (1.1% per year) and viscosity (1.3% per year), which together contribute to mechanical fragility [33]. The weakening of the ECM and the loss of its mechanical properties also compromise the structural support of dermal fibroblasts, accelerating their senescence and functional decline. Senescent fibroblasts, in turn, secrete matrix‐degrading enzymes and pro‐inflammatory cytokines that exacerbate ECM degradation, creating a self‐reinforcing loop that amplifies the skin aging process [34].

#### 3.1.2 Skin Hydration

Estrogen prevents age‐related dryness and maintains the skin's water‐holding capacity by stimulating the production of glycosaminoglycans such as hyaluronic acid [35, 36, 37, 38, 39]. These glycosaminoglycans attract water into the dermis through their negative ionic charge, promoting turgor and protecting against tissue compression while preserving the skin's suppleness [27]. Hypoestrogenism results in the loss of hydrophilic glycosaminoglycans, leading to a direct reduction in water content and skin suppleness [27].

**FIGURE 2:**
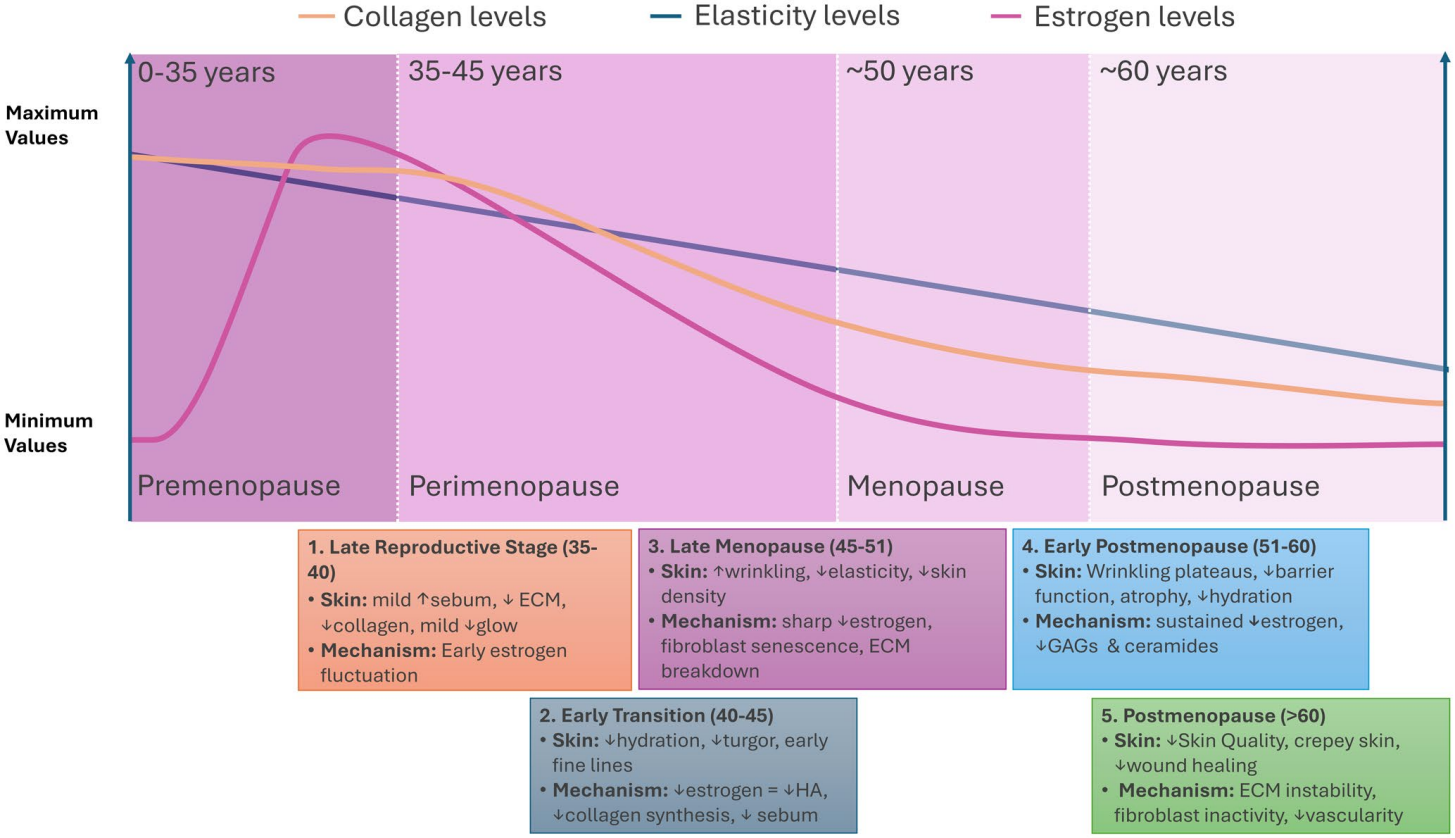
Illustrative trajectory of skin quality changes across menopause. A conceptual overview of skin quality changes across menopausal stages. Trajectories of collagen and elasticity are shown alongside other skin quality changes and proposed mechanistic drivers at each stage. Estrogen levels are depicted as peaking in the late 20s and declining gradually before menopause, with a sharper drop at the menopausal transition. Collagen and elasticity generally follow similar patterns. These trends represent average trajectories and may vary widely across individuals. ↑, increase; ↓, decrease; ECM, extracellular matrix; GAGs, glycosaminoglycans; HA, hyaluronic acid.

Sebum from sebaceous glands forms part of the skin's outermost barrier, helping minimize transepidermal water loss [39, 40]. During perimenopause, fluctuating estrogen and DHEA levels lead to inconsistent sebum production [8, 41]. Early in menopause, enlargement of the sebaceous glands provides temporary compensation for dryness, but as hypoestrogenism progresses, sebum production declines significantly, resulting in dry, sagging skin and atrophy (Figure 2) [8]. Postmenopausal sebum levels continue to decline gradually, with a 40% drop by the sixth decade and further reductions into the seventh, after which no major change occurs [42]. In contrast, men maintain relatively stable sebum production throughout life, with no significant decrease observed until the eighth decade [42].

#### 3.1.3 Wrinkling

Wrinkling results from the gradual deterioration of structural components in the dermis [39, 43]. Hypoestrogenism may accelerate this process through reduction in collagen, elastin, and hyaluronic acid, leading to increased skin rigidity and decreased elasticity [39]. In a cross‐sectional analysis of Korean women (*n* = 186), the risk of facial wrinkling increased significantly with years since menopause (OR = 3.909, 95% CI: 1.071–14.275) [44]. Additionally, women with a history of HRT had a significantly lower risk of facial wrinkling compared to those without HRT (OR = 0.211, 95% CI: 0.047–0.949) [44]. Histological and biomechanical analyses further demonstrate that postmenopausal skin is characterized by decreased epidermal thickness, increased stratum corneum, and reduced elasticity—all partially attributable to estrogen loss [13].

### 3.2 Impact of Hormone Replacement Therapy on Skin

HRT regimens typically include estrogen (17β‐estradiol in Europe or conjugated equine estrogen (CEE) in the United States), often combined with progestogens in women with an intact uterus to prevent endometrial hyperplasia [28]. Initial findings from the Women's Health Initiative in the early 2000s suggested that systemic HRT posed more risks than benefits for many women, contributing to a dramatic decline in its use—from nearly 40% of women in their 50s and 30% in their 60s in 2000 to just 7% in both age groups by 2010 [45]. This shift likely slowed progress in understanding the skin benefits of HRT. More recently, HRT has been found to offer a favorable benefit–risk ratio for alleviating vasomotor symptoms, genitourinary syndrome, and osteoporosis in women under 60 years of age or within 10 years of menopause onset, provided they have no contraindications [46, 47]. However, initiating HRT more than 10 years after menopause or after age 60 is associated with increased risks of coronary heart disease, stroke, venous thromboembolism, and dementia [46]. Although HRT appears to offer benefits for skin thickness, elasticity, and collagen when given near the onset of menopause, it is not currently indicated solely for treating estrogen‐deficient skin [21, 46].

To better understand estrogen's specific role in skin aging, many studies have compared postmenopausal women receiving HRT with those who do not, using both clinical and histological endpoints to assess changes in collagen, elasticity, and hydration [14]. Numerous early, small studies reported associations between HRT and improved skin characteristics via both systemic and topical administration [20, 48, 49, 50, 51, 52, 53, 54]. For example, in a randomized, double‐blind, placebo‐controlled trial of 60 postmenopausal French‐Canadian nuns—chosen to minimize variability in lifetime sun exposure, smoking, and diet—Maheux et al. [55] showed that 12 months of systemic CEE significantly increased the thickness of the skin by 11.5% (*p* < 0.01) and dermis thickness by 33% (*p* < 0.05), with no statistically significant difference observed in the control population. In a non‐randomized, placebo‐controlled study, Sauerbronn et al. [56] found a 6.49% increase in dermal collagen (*p* = 0.05) after 6 months of systemic HRT. In a third trial of 40 postmenopausal women aged 44–55, Sator et al. [57] demonstrated significant improvements in skin elasticity, thickness, and hydration after 7 months of oral 17β‐estradiol/dydrogesterone therapy, though skin surface lipids remained unchanged.

Another line of evidence comes from studies evaluating subcutaneous and local hormone delivery. Treatment with subcutaneous 17β‐estradiol and testosterone during the perimenopausal period was shown to prevent or reverse collagen loss [20]. Separate studies demonstrated that 17β‐estradiol treatment in postmenopausal women increased both type III collagen levels in the dermis, improving elasticity, and type I collagen, enhancing structural integrity [48, 49]. In an older patient cohort with established osteoporosis, 1 year of 17β‐estradiol treatment did not increase collagen content but enhanced collagen maturity by converting immature cross‐links into their mature form, thereby improving stability [50]. The authors suggested that collagen loss may become irreversible over time, or that a longer treatment duration may be needed to see benefits in older women [50]. Nonetheless, HRT‐induced stabilization strengthens the ECM, which is susceptible to deterioration in estrogen‐deficient skin [16, 27]. In some cases, such thinning could be restored by 30% with subcutaneous hormone therapy [20].

Despite these positive findings, not all studies have reported consistent effects. Low‐dose hormone therapy for 48 weeks in postmenopausal women did not significantly alter mild to moderate age‐related facial skin changes in a randomized, double‐blind, placebo‐controlled multicenter study [58]. Similarly, the KEEPS ancillary skin aging study, a 4‐year randomized, placebo‐controlled trial within the Kronos Early Estrogen Prevention Study, evaluated the effects of hormone therapy on facial wrinkles and skin rigidity in recently menopausal women [43]. The study found no statistically significant improvement in wrinkle scores or skin rigidity after 4 years of treatment with 17β‐estradiol or CEE compared to placebo, instead demonstrating skin color to be the strongest predictor of skin wrinkles and rigidity during the study period, with black women exhibiting the lowest total wrinkle scores across all time points compared to white women (*p* = 0.002). However, it is important to note that the KEEPS skin aging sub‐study acknowledged several design limitations, including the possibility of being underpowered, using relatively low HRT doses, and not incorporating a full range of skin assessments. The authors also acknowledged that a longer duration of treatment or a study incorporating both cosmetic and biochemical endpoints may be necessary to elucidate the role of HRT in age‐related skin conditions fully [43].

Although placebo‐controlled trial results on the effects of HRT on postmenopausal skin appear inconsistent (Table 1), large observational datasets also support beneficial skin effects of HRT [7, 39]. In a Japanese study of 176 postmenopausal women, skin elasticity declined at 0.55% per year, whereas HRT reversed this trend, resulting in a 5.2% gain over 12 months [19]. In another study, elasticity increased from approximately 40% to 60% in menopausal women receiving HRT (*n* = 75) compared to untreated controls (*n* = 75) [59]. In a separate study of 98 postmenopausal women who had received HRT for approximately 5 years, skin thickness increased by 7%–15% and sebum production increased by 35% compared to the untreated group [41]. Correspondingly, among menopausal women receiving HRT for at least 5 years, biophysical measurements were significantly higher for the parameters reflecting hydration and sebum secretion, which generally decrease after menopause [60]. Such positive findings were echoed in the National Health and Nutrition Examination Survey (NHANES I) of 3875 postmenopausal women, showing that estrogen use was associated with a statistically significant decrease in the likelihood of dry skin (odds ratio, 0.76; 95% confidence interval, 0.60–0.97) and wrinkling (odds ratio, 0.68; 95% confidence interval, 0.52–0.89) [61]. In contrast, 1‐year systemic HRT failed to alter the amount of collagen or the rate of synthesis in an open, non‐randomized parallel study of 43 early postmenopausal women. However, methods used may not have been sensitive enough to detect subtle qualitative changes in skin collagen, such as alterations in collagen cross‐linking or turnover rates, despite no observable quantitative changes [62].

**TABLE 1:** Placebo‐controlled studies comparing hormone replacement therapy (HRT) with no HRT in postmenopausal women (skin‐specific endpoints).

Study reference	*N*	Treatment	Measurements	Results
Castelo‐Branco et al., 1992 [53]	118	12 months of: Transdermal estrogen (*n* = 28) CEE continuously (*n* = 32) CEE cyclically (*n* = 28) Placebo (*n* = 30)	Skin collagen content (μg collagen/mg protein) at baseline and 12 months	Statistically significant differences between treated (Transdermal estrogen and CEE continuously) and control Transdermal estrogen group: +5.1% increase in collagen (*p* < 0.01) Continuous CEE group: +3.0% (*p* < 0.05) Cyclical CEE group: +1.8% (not statistically significant) Control: −3.2% (*p* < 0.05)
Meschia et al., 1994 [54]	40	1 year of: Transdermal estradiol patch (50 μg/day) + medroxyprogesterone acetate (*n* = 20) Placebo (*n* = 20)	Skin thickness measured radiologically	Increase in adjusted skin thickness in the HRT group compared to placebo Mean adjusted values: 0.98 mm (HRT) vs. 0.88 mm (placebo), (*p* = 0.0002)
Maheux et al., 1994 [55]	60	1 year of CEE (*n* = 30) Placebo (*n* = 30)	Thickness of the skin by ultrasonography Thickness of the dermis by skin biopsy Quality of life	Improvements were observed in women treated with HRT compared with those in the placebo group 11.5% increased skin thickness (*p* < 0.01) 33% increased dermis (*p* < 0.05) Enhanced quality of life (*p* < 0.05)
Sauerbronn et al., 2000 [56]	41	Cyclic scheme of 6 months of: Combination of valerate estradiol and cyproterone acetate (*n* = 21) Placebo (*n* = 20)	Histologic changes were evaluated by skin biopsy with computerized image analysis	Improvements were observed in women treated with HRT compared with those in the placebo group 6.49% increase in skin collagen (*p* = 0.05) No significant difference in thickness of epidermis, thickness of keratin, and elastic fibers content
Sator et al., 2007 [57]	40	Cyclic scheme of 7 months of: 17β‐estradiol/dydrogesterone (*n* = 20) Placebo (*n* = 20)	Skin elasticity Skin surface lipids Skin hydration Skin thickness Adverse‐event profile Clinical‐dermatological status	Improvements were observed in women treated with HRT compared with those in the placebo group Gross elasticity, net elasticity and portion of elasticity improvements were observed in women treated with HRT compared with those in the placebo group increased from 58% to 64% (*p* = 0.006), 48% to 63% (*p* = 0.002), and 35% to 39% (*p* = 0.005), respectively No significant changes in skin surface lipids, skin hydration Clinical‐dermatological status indicated increased turgor, moisture content, sebum production, and vascularization
Phillips et al., 2008 [58]	485	4 years of: 1 mg norethindrone acetate/5 μg and ethinyl estradiol (*n* = 162) 1 mg norethindrone acetate/10 μg and ethinyl estradiol (*n* = 158) Placebo (*n* = 165)	Coarse and fine facial wrinkling Subjective self‐assessment of changes Investigator global assessment of skin laxity/sagging, texture/dryness Patient self‐assessment of laxity/sagging, texture/dryness, and wrinkle depth Skin elasticity determined by timed deformation and recoil	No significant changes in postmenopausal women
Owen et al., 2016 [43]	116	4 years of: CEE (*n* = 38) Transdermal E2 with micronized P (*n* = 34) Placebo (*n* = 44)	Skin wrinkles were assessed at 11 locations on the face and neck Skin rigidity was assessed at the forehead and cheek	No significant changes in wrinkle score nor total rigidity score in early postmenopausal women

*Note:* Table includes placebo‐controlled trials reporting objective skin‐related outcomes in postmenopausal women receiving HRT that were identified in the literature search.

In addition to systemic therapy, several studies have explored the role of topical estrogen in mitigating skin aging. Topical formulations allow for localized effects on skin collagen, hydration, and elasticity with minimal systemic absorption, acting mainly through ER‐β, which is more prevalent in cutaneous tissues [63]. In a review by Rzepecki et al. of 23 studies on the effects of topical estrogen on postmenopausal skin, many studies reported positive impacts on menopausal skin characteristics; though variations in study design, application methods, estrogen concentration, concurrent systemic therapy, and control groups limited the ability to draw definitive conclusions. Most studies observed no systemic effects from topical application, but a few reported conflicting results. The authors concluded that topical estrogen may improve skin dryness, texture, elasticity, and reduce wrinkles in estrogen‐deficient skin; though further research is needed to establish clear treatment strategies [63]. More specifically, Varila et al. [49] reported a significant increase in collagen synthesis in the dermis after 3 months of topical estradiol versus placebo (*p* = 0.024). Masuda et al. [64] observed significant improvements in fine wrinkles and 3D topographic imaging after 4 weeks of topical estradiol (*p* < 0.01). A clinical study by Punnonen et al. specifically looked at the weaker potency estrogen, estriol. Topical application led to thickening of the papillary dermis and improved elastic fiber organization in postmenopausal skin, contributing to improved firmness and surface evenness [51].

Newer non‐hormonal estrogens such as methyl estradiolpropanoate (MEP) have also emerged as promising skin‐targeted agents. In a pilot study, Draelos [65] found that MEP significantly improved investigator‐assessed dryness, laxity, atrophy, and dullness (all *p* ≤ 0.003), as well as subject‐reported fine lines (*p* = 0.014) over 14 weeks without systemic absorption. Cohen [66] later confirmed improvements in hydration, texture, laxity, keratoses, and overall facial appearance, such as skin glow and tone, in a 20‐week open‐label trial using an MEP‐based regimen. A more recent study by Cohen and Downie [67] reported a 21% GAIS improvement in the periorbital area of postmenopausal women using the same MEP‐based regimen. These studies remain preliminary, with incompletely understood mechanisms of action and modest reported improvements, which require confirmation in larger, controlled trials. Nonetheless, they offer potential alternatives for women seeking skin improvements without systemic estrogen exposure.

A recent small study evaluated how menopause and HRT influence epidermal and dermal aging markers, focusing on skin structure and biomechanical function [13]. The study examined pre‐menopausal (*n* = 7), post‐menopausal (*n* = 11), and post‐menopausal women on HRT (*n* = 10), with age‐matched men for comparison (*n* = 29). In post‐menopausal women, epidermal thickness decreased, whereas stratum corneum thickness increased, indicating disrupted epidermal homeostasis [13]. Post‐menopausal women also showed reduced skin elasticity and increased viscoelasticity, leading to greater skin distension and structural and mechanical properties partially restored by HRT [13]. Furthermore, histological analysis of postmenopausal skin showed a reduction in CD44 expression, a receptor for hyaluronic acid essential for skin hydration and homeostasis [13]. This reduction was accompanied by reduced ceramide levels and increased cholesterol, indicating that alterations in lipid synthesis may contribute to epidermal barrier function. Interestingly, the study found lower estrogen levels correlated with higher viscoelasticity and reduced elasticity in women, suggesting prolonged skin “creep” or stretch after initial deformation. All menopause‐related changes were susceptible to regulation by HRT [13]. In contrast, no age‐related changes in elasticity were observed in the male cohort [13].

### 3.3 Aesthetic Treatments for Menopausal Women

Searches for minimally invasive aesthetic treatments targeting menopausal women revealed a gap in studies that intentionally collect demographic data on menopausal status or HRT use. Most existing research focuses on topical treatments, nutraceuticals, or genitourinary aesthetic procedures. Only one study specifically assessed menopausal status in the context of minimally invasive aesthetic treatments, reporting that temple volumization with HA fillers demonstrated good longevity and high patient satisfaction in both pre‐ and postmenopausal cohorts [68].

## 4 Discussion

Understanding the trajectory of estrogen‐related skin aging is increasingly relevant as perimenopausal and postmenopausal women comprise a large and growing demographic seeking aesthetic treatments [47]. Despite observational and clinical evidence showing hormone‐related skin changes, estrogen‐deficient skin remains an underrecognized clinical concern with no formal treatment indication. This review identifies gaps in robust evidence linking estrogen loss to skin aging that, if addressed, could inform aesthetic strategies. Given that many aesthetic interventions aim to reverse signs of aging associated with estrogen decline, it is essential to understand how time in menopause and duration of HRT therapy impact treatment response. Changes in treatment parameters and timing may be needed to ensure satisfactory outcomes in this population.

Notably, there is a clinical precedent for localized hormone use; for example, ACOG's 2021 guidance supports low‐dose vaginal estrogen for treatment of genitourinary symptoms following shared decision‐making [69]. Some dermatologists may similarly prescribe topical hormone creams, and products such as Alloy's topical estriol cream are available by prescription and supported by preliminary clinical evidence. However, peer‐reviewed and FDA‐reviewed data on their safety and efficacy in facial applications remain limited. Thus, the absence of a specific skin indication and regulatory approval is likely due to a lack of robust, peer‐reviewed clinical data assessing the specific benefit–risk ratio of HRT for skin, unlike the data available for other menopausal symptoms for which HRT is recommended [46]. The gap in recent, high‐quality evidence identified in our literature search is consistent with findings from a meta‐analysis by Pivazyan et al. [70], which aimed to evaluate the effects of menopausal hormone therapy on skin aging and identified only 15 eligible trials, none published in the past decade. This underscores the absence of robust, up‐to‐date studies evaluating HRT's impact on skin aging, leaving its skin benefits secondary and limited to women using HRT to manage other bothersome menopausal symptoms such as vasomotor symptoms or genitourinary syndrome [21]. In fact, many women are unaware of the physical changes associated with menopause, with 47% of postmenopausal women reporting not being informed about its effects on their skin, hair, or nails [8]. More than 50% of women partially or fully agreed that menopause has negatively affected their self‐esteem, yet only a quarter to half of them seek help for any menopausal symptoms [8, 71, 72]. Even when they do, they often encounter unclear referral pathways and limited menopause care from primary providers [47]. These unmet needs highlight the need for robust, well‐designed clinical studies to assess the impact of HRT on estrogen‐deficient skin, including comprehensive safety analyses, assessments of psychological outcomes, and determination of the optimal timing and duration of treatment to inform regulatory guidelines and drive changes in clinical practice.

Stronger clinical recommendations and more deliberate patient education are needed. Menopause‐related skin concerns are often dismissed as purely aesthetic rather than medical, despite their psychological impact, highlighting the need for a shift in clinical priorities to address skin health with the same importance as other menopause‐related issues [28]. Addressing skin quality is essential for overall wellness as well as enhancing the outcomes of facial aesthetic treatments such as fillers and facelifts [8, 9, 10]. Approximately one‐third of adults with aesthetic concerns are increasingly focused on skin quality, yet this issue is often overlooked by medical professionals [73]. To support this shift, more robust trials and further mechanistic insights are required, particularly regarding the role of ER‐β in dermal regulation and the localized effects of glycosaminoglycan loss. These insights may help explain why topical and non‐hormonal estrogens targeting these pathways have shown early promise in improving skin quality without systemic exposure [63, 65, 66]. Incorporating objective measures of skin quality, such as elasticity mapping, hydration indices, and standardized wrinkle scoring, alongside validated patient‐reported outcomes, could further strengthen the evidence base and support regulatory or guideline development.

In addition, future trials should also strive to include diverse skin types and ethnicities to ensure findings are applicable to global patient populations, especially given documented differences in post‐menopausal skin quality across skin colors [43, 74]. For example, Wolff et al. reported that recently menopausal Black women exhibited fewer facial wrinkles and greater skin rigidity compared to White women at baseline, highlighting intrinsic differences in skin structure and aging trajectories. Similarly, Owen et al. emphasized the need for more racially inclusive studies after observing that skin color was a significant predictor of wrinkle severity. These differential findings highlight the importance of studying diverse patient populations to fully characterize estrogen‐related skin aging.

Outside of HRT for skin quality and aesthetic concerns, dermal fillers are widely used to restore volume loss and correct altered facial contours among menopausal women [24, 28, 75]. Combining these interventions with strategies that promote sustained improvements by supporting endogenous collagen, elastin, and hyaluronic acid synthesis may offer more durable aesthetic and functional outcomes for postmenopausal women [28]. Our literature review revealed that few aesthetic studies intentionally collect demographic data on menopausal status or HRT use. The value of gathering this information is demonstrated by Müller et al. [68], where similar techniques for temple volumization with HA filler showed good longevity and high patient satisfaction in both pre‐ and postmenopausal cohorts. These results highlight valuable clinical strategies that physicians can reference when addressing postmenopausal aesthetic concerns. However, cosmetic treatments that rely on normal fibroblast function to produce skin components and facilitate healing—such as energy‐based devices and biostimulatory fillers—have not been studied to determine whether outcomes differ among perimenopausal, menopausal, and HRT‐treated menopausal patients, to our knowledge. We recommend that future research be more deliberate in analyzing patient cohorts based on menopausal and HRT status, especially given that in 2023, patients aged 40–69 accounted for over two‐thirds of cosmetic surgeries and more than three‐quarters of minimally invasive procedures performed in the United States [76].

In addition to HRT, several aesthetic and therapeutic interventions show promise for addressing the structural and functional changes associated with postmenopausal skin. Hyper‐diluted calcium hydroxyapatite (CaHA; Radiesse, Merz Aesthetics, Raleigh, North Carolina), typically used as a dermal filler, has demonstrated benefits when diluted with lidocaine or saline, stimulating collagen and elastin synthesis and increasing neovascularization without a volumizing effect [77]. This technique has been associated with improvements in dermal thickness, elasticity, and pliability, particularly in the neck and décolletage [77]. Similarly, microfocused ultrasound (MFU‐V) offers an effective non‐invasive approach for tightening and lifting lax skin [78]. By targeting the SMAS and platysma layers, MFU‐V induces neocollagenesis and collagen remodeling, helping to restore skin firmness [78, 79]. A recent randomized clinical study supports the combination of MFU‐V and CaHA for age‐related skin changes, showing significant improvements in marionette lines, jawline contour, and neck appearance after 15 months. The treatments enhanced collagen and elastin production, stimulated neoangiogenesis, and remodeled superficial and deep dermal layers, demonstrating their synergistic effects [80]. Notably, MFU‐V has shown comparable effectiveness in older patients, with those over 60 achieving similar improvements in skin laxity as younger patients, despite reduced collagen levels [78, 81]. More prospective studies focused on postmenopausal women will be essential to refine treatment strategies, including combinations of aesthetic treatment modalities with or without HRT as well as determining optimal doses or treatment frequencies, to equip physicians with options for managing this growing patient population.

## 5 Conclusion

The steep decline in estrogen levels during menopause drives marked changes in skin quality, leading to decreased collagen, elasticity, and moisture. Many women remain uninformed about menopause's impact on their skin, which may affect their self‐esteem and psychological well‐being. Although HRT can alleviate these effects, its approved indications for menopausal symptoms do not extend to treating estrogen‐deficient skin due to insufficient data demonstrating a favorable benefit–risk balance. Furthermore, combining HRT with aesthetic interventions may enhance collagen synthesis and improve skin thickness, elasticity, and hydration. More robust clinical studies are needed that intentionally collect and analyze data on menopausal and HRT status to refine treatment strategies and enable physicians to provide their patients with more effective care. Given the rising demand for cosmetic procedures among women aged 40–69, integrating skin health for peri‐ and postmenopausal women is becoming increasingly important [76]. Moving forward, addressing menopausal skin concerns with the same priority as other menopause‐related health issues may facilitate better care and improved quality of life for postmenopausal women.

## Author Contributions

All authors made equally significant contributions to the concept, design, and execution of this manuscript.

## Ethics Statement

The authors have nothing to report.

## Conflicts of Interest

The authors declare no conflicts of interest.

## Data Availability

The authors have nothing to report.
